# Supplementary material for: Exercise Dose Equalization in High-Intensity Interval Training: A Scoping Review
Source: Int J Environ Res Public Health. 2022 Apr 20;19(9):4980. doi: 10.3390/ijerph19094980 (PMC9104727; doi:10.3390/ijerph19094980)
Supplement: Supplementary file 1 [file ijerph-19-04980-s001.zip › Supplementary File S2.pdf]

Supplementary File S2. Checklist for the assessment of the methodological quality for HIIT studies, adapted from Downs and Black (1998).

1- Were the patients in different intervention groups (trials and cohort studies) or were the cases and controls (case-control studies) recruited from the same population?

The proportion of those asked who agreed should be stated. Validation that the sample was representative would include demonstrating that the distribution of the main confounding factors was the same in the study sample and the source population.

2- Were study subjects randomized to intervention groups?

Studies which state that subjects were randomized should be answered yes except where method of randomization would not ensure random allocation. For example, alternate allocation would score no because it is predictable.

3- Were intervention and control groups matched for physical capacities relevant for the training protocol?

4- Were training sessions directly supervised by investigators or coachs?

If exercise was performed free or at home, the question should be answered no.

5- Were exercise variables monitored and controlled throughout the sessions ?

If any tool or technology was used, the question should be answered yes.

6- Was adherence to training monitored, even after sessions ?

Where there was non-adherence with the training or not reported, the question should be answered no.

7- Were losses of patients to follow-up taken into account?

If the numbers of patients lost to follow-up are not reported, the question should be answered as unable to determine. If the proportion lost to follow-up was too small to affect the main findings, the question should be answered yes.

8- Did the study have sufficient power to detect a clinically important effect where the probability value for a difference being due to chance is less than 5%?

Sample sizes have been calculated to detect a difference of x% and y%.
