# Supplementary material for: Exercise Dose Equalization in High-Intensity Interval Training: A Scoping Review
Source: Int J Environ Res Public Health. 2022 Apr 20;19(9):4980. doi: 10.3390/ijerph19094980 (PMC9104727; doi:10.3390/ijerph19094980)
Supplement: Supplementary file 1 [file ijerph-19-04980-s001.zip › Supplementary File S3.pdf]

1. *Articles without protocols equalization*

1 Abdelbasset WK, Tantawy SA, Kamel DM, et al. Effects of high-intensity interval and moderate-intensity continuous aerobic exercise on diabetic obese patients with nonalcoholic fatty liver disease: A comparative randomized controlled trial. *Medicine (Baltimore)* 2020; 99(10):e19471. Doi: 10.1097/MD.0000000000019471.

2 Almenning I, Rieber-Mohn A, Lundgren KM, et al. Effects of High Intensity Interval Training and Strength Training on Metabolic, Cardiovascular and Hormonal Outcomes in Women with Polycystic Ovary Syndrome: A Pilot Study. *PLOS ONE* 2015; 10(9):e0138793. Doi: 10.1371/journal.pone.0138793.

3 Amaro-Gahete FJ, De-la-O A, Jurado-Fasoli L, et al. Exercise training increases the S-Klotho plasma levels in sedentary middle-aged adults: A randomised controlled trial. The FIT-AGEING study. *J Sports Sci* 2019; 37(19):2175–2183. Doi: 10.1080/02640414.2019.1626048.

4 Angadi SS, Mookadam F, Lee CD, et al. High-intensity interval training vs. moderate-intensity continuous exercise training in heart failure with preserved ejection fraction: a pilot study. *J Appl Physiol (1985)* 2015; 119(6):753–758. Doi: 10.1152/japplphysiol.00518.2014.

5 Arboleda-Serna VH, Feito Y, Patiño-Villada FA, et al. Effects of high-intensity interval training compared to moderate-intensity continuous training on maximal oxygen consumption and blood pressure in healthy men: A randomized controlled trial. *Biomedica* 2019; 39(3):524–536. Doi: 10.7705/biomedica.4451.

6 Ballesta-García I, Martínez-González-Moro I, Rubio-Arias JÁ, et al. High-Intensity Interval Circuit Training Versus Moderate-Intensity Continuous Training on Functional Ability and Body Mass Index in Middle-Aged and Older Women: A Randomized Controlled Trial. *Int J Environ Res Public Health* 2019; 16(21):E4205. Doi: 10.3390/ijerph16214205.

7 Benda NMM, Seeger JPH, Stevens GGCF, et al. Effects of High-Intensity Interval Training versus Continuous Training on Physical Fitness, Cardiovascular Function and Quality of Life in Heart Failure Patients. *PLoS One* 2015; 10(10):e0141256. Doi: 10.1371/journal.pone.0141256.

8 Besnier F, Labrunée M, Richard L, et al. Short-term effects of a 3-week interval training program on heart rate variability in chronic heart failure. A randomised controlled trial. *Ann Phys Rehabil Med* 2019; 62(5):321–328. Doi: 10.1016/j.rehab.2019.06.013.

9 Breil FA, Weber SN, Koller S, et al. Block training periodization in alpine skiing: effects of 11-day HIT on VO2max and performance. *Eur J Appl Physiol* 2010; 109(6):1077–1086. Doi: 10.1007/s00421-010-1455-1.

10 Bruseghini P, Tam E, Calabria E, et al. High Intensity Interval Training Does Not Have Compensatory Effects on Physical Activity Levels in Older Adults. *International Journal of Environmental Research and Public Health* 2020; 17:1083. Doi: 10.3390/ijerph17031083.

11 Camacho-Cardenosa A, Camacho-Cardenosa M, Brazo-Sayavera J, et al. Effects of High-Intensity Interval Training Under Normobaric Hypoxia on Cardiometabolic Risk Markers in Overweight/Obese Women. *High Alt Med Biol* 2018; 19(4):356–366. Doi: 10.1089/ham.2018.0059.

12 Chuensiri N, Suksom D, Tanaka H. Effects of High-Intensity Intermittent Training on Vascular Function in Obese Preadolescent Boys. *Child Obes* 2018; 14(1):41–49. Doi: 10.1089/chi.2017.0024.

13 Cilindro C, Gholamalishahi S, La Torre G, et al. Efficacy of the curves training program for loosing body weight, body circumferences and fat mass percentage: a non randomized clinical trial. *Clin Ter* 2019; 170(4):e235–e240. Doi: 10.7417/CT.2019.2139.

14 Cox ER, Gajanand T, Burton NW, et al. Effect of different exercise training intensities on musculoskeletal and neuropathic pain in inactive individuals with type 2 diabetes - Preliminary randomised controlled trial. *Diabetes Res Clin Pract* 2020; 164:108168. Doi: 10.1016/j.diabres.2020.108168.

15 Currie KD, Dubberley JB, McKELVIE RS, et al. Low-Volume, High-Intensity Interval Training in Patients with CAD. *Medicine & Science in Sports & Exercise* 2013; 45(8):1436–1442. Doi: 10.1249/MSS.0b013e31828bbbd4.

16 Cvetković N, Stojanović E, Stojiljković N, et al. Exercise training in overweight and obese children: Recreational football and high-intensity interval training provide similar benefits to physical fitness. *Scand J Med Sci Sports* 2018; 28 Suppl 1:18–32. Doi: 10.1111/sms.13241.

17 Dall CH, Snoer M, Christensen S, et al. Effect of high-intensity training versus moderate training on peak oxygen uptake and chronotropic response in heart transplant recipients: a randomized crossover trial. *Am J Transplant* 2014; 14(10):2391–2399. Doi: 10.1111/ajt.12873.

18 Dellal A, Varliette C, Owen A, et al. Small-sided games versus interval training in amateur soccer players: effects on the aerobic capacity and the ability to perform intermittent exercises with changes of direction. *J Strength Cond Res* 2012; 26(10):2712–2720. Doi: 10.1519/JSC.0b013e31824294c4.

19 Devin JL, Jenkins DG, Sax AT, et al. Cardiorespiratory Fitness and Body Composition Responses to Different Intensities and Frequencies of Exercise Training in Colorectal Cancer Survivors. *Clin Colorectal Cancer* 2018; 17(2):e269–e279. Doi: 10.1016/j.clcc.2018.01.004.

20 Dunham C, Harms CA. Effects of high-intensity interval training on pulmonary function. *Eur J Appl Physiol* 2012; 112(8):3061–3068. Doi: 10.1007/s00421-011-2285-5.

21 Farinha JB, Ramis TR, Vieira AF, et al. Glycemic, inflammatory and oxidative stress responses to different high-intensity training protocols in type 1 diabetes: A randomized clinical trial. *J Diabetes Complications* 2018; 32(12):1124–1132. Doi: 10.1016/j.jdiacomp.2018.09.008.

22 Farley ORL, Secomb JL, Parsonage JR, et al. Five Weeks of Sprint and High-Intensity

Interval Training Improves Paddling Performance in Adolescent Surfers. *J Strength Cond Res* 2016; 30(9):2446–2452. Doi: 10.1519/JSC.0000000000001364.

23 Faude O, Schnittker R, Schulte-Zurhausen R, et al. High intensity interval training vs. high-volume running training during pre-season conditioning in high-level youth football: a cross-over trial. *J Sports Sci* 2013; 31(13):1441–1450. Doi: 10.1080/02640414.2013.792953.

24 Fernandez-Fernandez J, Zimek R, Wiewelhove T, et al. High-intensity interval training vs. repeated-sprint training in tennis. *J Strength Cond Res* 2012; 26(1):53–62. Doi: 10.1519/JSC.0b013e318220b4ff.

25 Ferrari Bravo D, Impellizzeri FM, Rampinini E, et al. Sprint vs. interval training in football. *Int J Sports Med* 2008; 29(8):668–674. Doi: 10.1055/s-2007-989371.

26 Flaherty JM, Smoliga JM, Zavorsky GS. The effect of increased physical activity on pulmonary diffusing capacity in unfit women. *Exp Physiol* 2014; 99(3):562–570. Doi: 10.1113/expphysiol.2013.076406.

27 Hajizadeh Maleki B, Tartibian B, Chehrazi M. The effects of three different exercise modalities on markers of male reproduction in healthy subjects: a randomized controlled trial. *Reproduction* 2017; 153(2):157–174. Doi: 10.1530/REP-16-0318.

28 Hollekim-Strand SM, Høydahl SF, Follestad T, et al. Exercise Training Normalizes Timing of Left Ventricular Untwist Rate, but Not Peak Untwist Rate, in Individuals with Type 2 Diabetes and Diastolic Dysfunction: A Pilot Study. *J Am Soc Echocardiogr* 2016; 29(5):421-430.e2. Doi: 10.1016/j.echo.2016.01.005.

29 Horak M, Zlamal F, Iliev R, et al. Exercise-induced circulating microRNA changes in athletes in various training scenarios. *PLoS One* 2018; 13(1):e0191060. Doi: 10.1371/journal.pone.0191060.

30 Inoue A, Impellizzeri FM, Pires FO, et al. Effects of Sprint versus High-Intensity Aerobic Interval Training on Cross-Country Mountain Biking Performance: A Randomized Controlled Trial. *PLoS One* 2016; 11(1):e0145298. Doi: 10.1371/journal.pone.0145298.

31 Jakobsen MD, Sundstrup E, Randers MB, et al. The effect of strength training, recreational soccer and running exercise on stretch-shortening cycle muscle performance during countermovement jumping. *Hum Mov Sci* 2012; 31(4):970–986. Doi: 10.1016/j.humov.2011.10.001.

32 Jorissen W, Vanmierlo T, Wens I, et al. Twelve Weeks of Medium-Intensity Exercise Therapy Affects the Lipoprotein Profile of Multiple Sclerosis Patients. *International Journal of Molecular Sciences* 2018; 19(1):193. Doi: 10.3390/ijms19010193.

33 Keating SE, Machan EA, O'Connor HT, et al. Continuous exercise but not high intensity interval training improves fat distribution in overweight adults. *J Obes* 2014; 2014:834865. Doi: 10.1155/2014/834865.

34 Klonizakis M, Moss J, Gilbert S, et al. Low-volume high-intensity interval training rapidly improves cardiopulmonary function in postmenopausal women. *Menopause*

2014; 21(10):1099–1105. Doi: 10.1097/GME.0000000000000208.
35 Koh H-CE, Ørtenblad N, Winding KM, et al. High-intensity interval, but not endurance, training induces muscle fiber type-specific subsarcolemmal lipid droplet size reduction in type 2 diabetic patients. *Am J Physiol Endocrinol Metab* 2018; 315(5):E872–E884. Doi: 10.1152/ajpendo.00161.2018.
36 Koufaki P, Mercer TH, George KP, et al. Low-volume high-intensity interval training vs continuous aerobic cycling in patients with chronic heart failure: a pragmatic randomised clinical trial of feasibility and effectiveness. *J Rehabil Med* 2014; 46(4):348–356. Doi: 10.2340/16501977-1278.
37 Mallard AR, Hollekim-Strand SM, Coombes JS, et al. Exercise intensity, redox homeostasis and inflammation in type 2 diabetes mellitus. *J Sci Med Sport* 2017; 20(10):893–898. Doi: 10.1016/j.jsams.2017.03.014.
38 Martinez-Valdes E, Farina D, Negro F, et al. Early Motor Unit Conduction Velocity Changes to High-Intensity Interval Training versus Continuous Training. *Med Sci Sports Exerc* 2018; 50(11):2339–2350. Doi: 10.1249/MSS.0000000000001705.
39 Martins FM, de Paula Souza A, Nunes PRP, et al. High-intensity body weight training is comparable to combined training in changes in muscle mass, physical performance, inflammatory markers and metabolic health in postmenopausal women at high risk for type 2 diabetes mellitus: A randomized controlled clinical trial. *Exp Gerontol* 2018; 107:108–115. Doi: 10.1016/j.exger.2018.02.016.
40 Matsuo T, So R, Shimojo N, et al. Effect of aerobic exercise training followed by a low-calorie diet on metabolic syndrome risk factors in men. *Nutr Metab Cardiovasc Dis* 2015; 25(9):832–838. Doi: 10.1016/j.numecd.2015.05.009.
41 Matsuo T, Saotome K, Seino S, et al. Effects of a low-volume aerobic-type interval exercise on VO2max and cardiac mass. *Med Sci Sports Exerc* 2014; 46(1):42–50. Doi: 10.1249/MSS.0b013e3182a38da8.
42 Matsuo T, Saotome K, Seino S, et al. Low-volume, high-intensity, aerobic interval exercise for sedentary adults: VO_2max, cardiac mass, and heart rate recovery. *Eur J Appl Physiol* 2014; 114(9):1963–1972. Doi: 10.1007/s00421-014-2917-7.
43 McKay BR, Paterson DH, Kowalchuk JM. Effect of short-term high-intensity interval training vs. continuous training on O2 uptake kinetics, muscle deoxygenation, and exercise performance. *J Appl Physiol (1985)* 2009; 107(1):128–138. Doi: 10.1152/japplphysiol.90828.2008.
44 McRae G, Payne A, Zelt JGE, et al. Extremely low volume, whole-body aerobic-resistance training improves aerobic fitness and muscular endurance in females. *Appl Physiol Nutr Metab* 2012; 37(6):1124–1131. Doi: 10.1139/h2012-093.
45 Morales-Palomo F, Ramirez-Jimenez M, Ortega JF, et al. Effectiveness of Aerobic Exercise Programs for Health Promotion in Metabolic Syndrome. *Med Sci Sports Exerc* 2019; 51(9):1876–1883. Doi: 10.1249/MSS.0000000000001983.
46 Morrissey C, Montero D, Raverdy C, et al. Effects of Exercise Intensity on

Microvascular Function in Obese Adolescents. *Int J Sports Med* 2018; 39(6):450–455. Doi: 10.1055/a-0577-4280.
47 Mortensen SP, Winding KM, Iepsen UW, et al. The effect of two exercise modalities on skeletal muscle capillary ultrastructure in individuals with type 2 diabetes. *Scand J Med Sci Sports* 2019; 29(3):360–368. Doi: 10.1111/sms.13348.
48 Northey JM, Pumpa KL, Quinlan C, et al. Cognition in breast cancer survivors: A pilot study of interval and continuous exercise. *J Sci Med Sport* 2019; 22(5):580–585. Doi: 10.1016/j.jsams.2018.11.026.
49 Nugent F, Comyns T, Nevill A, et al. The Effects of Low-Volume, High-Intensity Training on Performance Parameters in Competitive Youth Swimmers. *Int J Sports Physiol Perform* 2019; 14(2):203–208. Doi: 10.1123/ijspp.2018-0110.
50 Nunes PRP, Martins FM, Souza AP, et al. Effect of high-intensity interval training on body composition and inflammatory markers in obese postmenopausal women: a randomized controlled trial. *Menopause* 2019; 26(3):256–264. Doi: 10.1097/GME.0000000000001207.
51 Pagola I, Morales JS, Alejo LB, et al. Concurrent Exercise Interventions in Breast Cancer Survivors with Cancer-related Fatigue. *Int J Sports Med* 2020; 41(11):790–797. Doi: 10.1055/a-1147-1513.
52 Poon ET-C, Little JP, Sit CH-P, et al. The effect of low-volume high-intensity interval training on cardiometabolic health and psychological responses in overweight/obese middle-aged men. *J Sports Sci* 2020; 38(17):1997–2004. Doi: 10.1080/02640414.2020.1766178.
53 Reljic D, Wittmann F, Fischer JE. Effects of low-volume high-intensity interval training in a community setting: a pilot study. *Eur J Appl Physiol* 2018; 118(6):1153–1167. Doi: 10.1007/s00421-018-3845-8.
54 Robinson MM, Dasari S, Konopka AR, et al. Enhanced Protein Translation Underlies Improved Metabolic and Physical Adaptations to Different Exercise Training Modes in Young and Old Humans. *Cell Metab* 2017; 25(3):581–592. Doi: 10.1016/j.cmet.2017.02.009.
55 Rodrigues JAL, Ferrari GD, Trapé ÁA, et al. β2 adrenergic interaction and cardiac autonomic function: effects of aerobic training in overweight/obese individuals. *Eur J Appl Physiol* 2020; 120(3):613–624. Doi: 10.1007/s00421-020-04301-z.
56 Roy M, Williams SM, Brown RC, et al. High-Intensity Interval Training in the Real World: Outcomes from a 12-Month Intervention in Overweight Adults. *Med Sci Sports Exerc* 2018; 50(9):1818–1826. Doi: 10.1249/MSS.0000000000001642.
57 Runacres A, Mackintosh KA, McNarry MA. The effect of constant-intensity endurance training and high-intensity interval training on aerobic and anaerobic parameters in youth. *J Sports Sci* 2019; 37(21):2492–2498. Doi: 10.1080/02640414.2019.1644890.
58 Sawyer BJ, Tucker WJ, Bhammar DM, et al. Effects of high-intensity interval training and moderate-intensity continuous training on endothelial function and

cardiometabolic risk markers in obese adults. *J Appl Physiol (1985)* 2016; 121(1):279–288. Doi: 10.1152/japplphysiol.00024.2016.
59 Schmitt J, Lindner N, Reuss-Borst M, et al. A 3-week multimodal intervention involving high-intensity interval training in female cancer survivors: a randomized controlled trial. *Physiological Reports* 2016; 4(3):e12693. Doi: 10.14814/phy2.12693.
60 Schubert MM, Clarke HE, Seay RF, et al. Impact of 4 weeks of interval training on resting metabolic rate, fitness, and health-related outcomes. *Appl Physiol Nutr Metab* 2017; 42(10):1073–1081. Doi: 10.1139/apnm-2017-0268.
61 Scott SN, Cocks M, Andrews RC, et al. High-Intensity Interval Training Improves Aerobic Capacity Without a Detrimental Decline in Blood Glucose in People With Type 1 Diabetes. *J Clin Endocrinol Metab* 2019; 104(2):604–612. Doi: 10.1210/jc.2018-01309.
62 Scott S, Shepherd S, Hopkins N, et al. Home-HIT improves muscle capillarisation and eNOS/NAD(P)Hoxidase protein ratio in obese individuals with elevated cardiovascular disease risk. *The Journal of Physiology* 2019; 597. Doi: 10.1113/jp278062.
63 Sheykhlouvand M, Gharaat M, Khalili E, et al. Low-Volume High-Intensity Interval Versus Continuous Endurance Training: Effects on Hematological and Cardiorespiratory System Adaptations in Professional Canoe Polo Athletes. *J Strength Cond Res* 2018; 32(7):1852–1860. Doi: 10.1519/JSC.0000000000002112.
64 Sijie T, Hainai Y, Fengying Y, et al. High intensity interval exercise training in overweight young women. *J Sports Med Phys Fitness* 2012; 52(3):255–262.
65 Sperlich B, Zinner C, Heilemann I, et al. High-intensity interval training improves VO(2peak), maximal lactate accumulation, time trial and competition performance in 9-11-year-old swimmers. *Eur J Appl Physiol* 2010; 110(5):1029–1036. Doi: 10.1007/s00421-010-1586-4.
66 Stensvold D, Slørdahl SA, Wisløff U. Effect of exercise training on inflammation status among people with metabolic syndrome. *Metab Syndr Relat Disord* 2012; 10(4):267–272. Doi: 10.1089/met.2011.0140.
67 Streese L, Kotliar K, Deiseroth A, et al. Retinal endothelial function in cardiovascular risk patients: A randomized controlled exercise trial. *Scand J Med Sci Sports* 2020; 30(2):272–280. Doi: 10.1111/sms.13560.
68 Wens I, Dalgas U, Vandenabeele F, et al. High Intensity Aerobic and Resistance Exercise Can Improve Glucose Tolerance in Persons With Multiple Sclerosis: A Randomized Controlled Trial. *Am J Phys Med Rehabil* 2017; 96(3):161–166. Doi: 10.1097/PHM.0000000000000563.
69 Wouda MF, Lundgaard E, Becker F, et al. Effects of moderate- and high-intensity aerobic training program in ambulatory subjects with incomplete spinal cord injury-a randomized controlled trial. *Spinal Cord* 2018; 56(10):955–963. Doi: 10.1038/s41393-018-0140-9.
70 Zimmer P, Bloch W, Schenk A, et al. High-intensity interval exercise improves cognitive performance and reduces matrix metalloproteinases-2 serum levels in

persons with multiple sclerosis: A randomized controlled trial. *Mult Scler* 2018; 24(12):1635–1644. Doi: 10.1177/1352458517728342.

71 Zinner C, Schäfer Olstad D, Sperlich B. Mesocycles with Different Training Intensity Distribution in Recreational Runners. *Med Sci Sports Exerc* 2018; 50(8):1641–1648. Doi: 10.1249/MSS.0000000000001599.

2. Articles with protocols equalization

2.1. Equalization by energy-based methods

1 Ahmadizad S, Avansar AS, Ebrahim K, et al. The effects of short-term high-intensity interval training vs. moderate-intensity continuous training on plasma levels of nesfatin-1 and inflammatory markers. *Horm Mol Biol Clin Investig* 2015; 21(3):165–173. Doi: 10.1515/hmbci-2014-0038.

2 Bækkerud FH, Solberg F, Leinan IM, et al. Comparison of Three Popular Exercise Modalities on V˙O2max in Overweight and Obese. *Med Sci Sports Exerc* 2016; 48(3):491–498. Doi: 10.1249/MSS.0000000000000777.

3 Beetham KS, Howden EJ, Fassett RG, et al. High-intensity interval training in chronic kidney disease: A randomized pilot study. *Scand J Med Sci Sports* 2019; 29(8):1197–1204. Doi: 10.1111/sms.13436.

4 Berger NJA, Tolfrey K, Williams AG, et al. Influence of continuous and interval training on oxygen uptake on-kinetics. *Med Sci Sports Exerc* 2006; 38(3):504–512. Doi: 10.1249/01.mss.0000191418.37709.81.

5 Bottoms L, Leighton D, Carpenter R, et al. Affective and enjoyment responses to 12 weeks of high intensity interval training and moderate continuous training in adults with Crohn's disease. *PLoS One* 2019; 14(9):e0222060. Doi: 10.1371/journal.pone.0222060.

6 Castro A, Duft RG, Ferreira MLV, et al. Association of skeletal muscle and serum metabolites with maximum power output gains in response to continuous endurance or high-intensity interval training programs: The TIMES study - A randomized controlled trial. *PLoS One* 2019; 14(2):e0212115. Doi: 10.1371/journal.pone.0212115.

7 Coetsee C, Terblanche E. Cerebral oxygenation during cortical activation: the differential influence of three exercise training modalities. A randomized controlled trial. *Eur J Appl Physiol* 2017; 117(8):1617–1627. Doi: 10.1007/s00421-017-3651-8.

8 Coletta AM, Brewster AM, Chen M, et al. High-Intensity Interval Training Is Feasible in Women at High Risk for Breast Cancer. *Med Sci Sports Exerc* 2019; 51(11):2193–2200. Doi: 10.1249/MSS.0000000000002048.

9 Cunningham DA, McCrimmon D, Vlach LF. Cardiovascular response to interval and continuous training in women. *Eur J Appl Physiol Occup Physiol* 1979; 41(3):187–197. Doi: 10.1007/BF00430011.

10 Dall CH, Gustafsson F, Christensen SB, et al. Effect of moderate- versus high-intensity exercise on vascular function, biomarkers and quality of life in heart

transplant recipients: A randomized, crossover trial. *J Heart Lung Transplant* 2015; 34(8):1033–1041. Doi: 10.1016/j.healun.2015.02.001.
11 de Lira CT, Dos Santos MA, Gomes PP, et al. Aerobic training performed at ventilatory threshold improves liver enzymes and lipid profile related to non-alcoholic fatty liver disease in adolescents with obesity. *Nutr Health* 2017; 23(4):281–288. Doi: 10.1177/0260106017720350.
12 Dias KA, Ingul CB, Tjønna AE, et al. Effect of High-Intensity Interval Training on Fitness, Fat Mass and Cardiometabolic Biomarkers in Children with Obesity: A Randomised Controlled Trial. *Sports Med* 2018; 48(3):733–746. Doi: 10.1007/s40279-017-0777-0.
13 Drigny J, Gremeaux V, Guiraud T, et al. Long-term high-intensity interval training associated with lifestyle modifications improves QT dispersion parameters in metabolic syndrome patients. *Ann Phys Rehabil Med* 2013; 56(5):356–370. Doi: 10.1016/j.rehab.2013.03.005.
14 Driller MW, Fell JW, Gregory JR, et al. The effects of high-intensity interval training in well-trained rowers. *Int J Sports Physiol Perform* 2009; 4(1):110–121. Doi: 10.1123/ijspp.4.1.110.
15 Dun Y, Thomas RJ, Smith JR, et al. High-intensity interval training improves metabolic syndrome and body composition in outpatient cardiac rehabilitation patients with myocardial infarction. *Cardiovasc Diabetol* 2019; 18(1):104. Doi: 10.1186/s12933-019-0907-0.
16 Dünnwald T, Melmer A, Gatterer H, et al. Supervised Short-term High-intensity Training on Plasma Irisin Concentrations in Type 2 Diabetic Patients. *Int J Sports Med* 2019; 40(3):158–164. Doi: 10.1055/a-0828-8047.
17 Edge J, Bishop D, Goodman C, et al. Effects of high- and moderate-intensity training on metabolism and repeated sprints. *Med Sci Sports Exerc* 2005; 37(11):1975–1982. Doi: 10.1249/01.mss.0000175855.35403.4c.
18 Ellingsen Ø, Halle M, Conraads V, et al. High-Intensity Interval Training in Patients With Heart Failure With Reduced Ejection Fraction. *Circulation* 2017; 135(9):839–849. Doi: 10.1161/CIRCULATIONAHA.116.022924.
19 Fyfe JJ, Bishop DJ, Bartlett JD, et al. Enhanced skeletal muscle ribosome biogenesis, yet attenuated mTORC1 and ribosome biogenesis-related signalling, following short-term concurrent versus single-mode resistance training. *Sci Rep* 2018; 8(1):560. Doi: 10.1038/s41598-017-18887-6.
20 Gerosa-Neto J, Panissa VLG, Monteiro PA, et al. High- or moderate-intensity training promotes change in cardiorespiratory fitness, but not visceral fat, in obese men: A randomised trial of equal energy expenditure exercise. *Respir Physiol Neurobiol* 2019; 266:150–155. Doi: 10.1016/j.resp.2019.05.009.
21 Granata C, S.F. Oliveira R, Little J, et al. Training intensity modulates changes in PGC-1 and p53 protein content and mitochondrial respiration, but not markers of

mitochondrial content in human skeletal muscle. *FASEB Journal : Official Publication of the Federation of American Societies for Experimental Biology* 2015; 30. Doi: 10.1096/fj.15-276907.
22 Hakansson S, Jones MD, Ristov M, et al. Intensity-dependent effects of aerobic training on pressure pain threshold in overweight men: A randomized trial. *Eur J Pain* 2018; 22(10):1813–1823. Doi: 10.1002/ejp.1277.
23 Hanssen H, Minghetti A, Magon S, et al. Effects of different endurance exercise modalities on migraine days and cerebrovascular health in episodic migraineurs: A randomized controlled trial. *Scand J Med Sci Sports* 2018; 28(3):1103–1112. Doi: 10.1111/sms.13023.
24 Heiston EM, Eichner NZM, Gilbertson NM, et al. Two weeks of exercise training intensity on appetite regulation in obese adults with prediabetes. *J Appl Physiol (1985)* 2019; 126(3):746–754. Doi: 10.1152/japplphysiol.00655.2018.
25 Heisz JJ, Tejada MGM, Paolucci EM, et al. Enjoyment for High-Intensity Interval Exercise Increases during the First Six Weeks of Training: Implications for Promoting Exercise Adherence in Sedentary Adults. *PLoS One* 2016; 11(12):e0168534. Doi: 10.1371/journal.pone.0168534.
26 Helgerud J, Høydal K, Wang E, et al. Aerobic high-intensity intervals improve VO2max more than moderate training. *Med Sci Sports Exerc* 2007; 39(4):665–671. Doi: 10.1249/mss.0b013e3180304570.
27 Hwang C-L, Lim J, Yoo J-K, et al. Effect of all-extremity high-intensity interval training vs. moderate-intensity continuous training on aerobic fitness in middle-aged and older adults with type 2 diabetes: A randomized controlled trial. *Exp Gerontol* 2019; 116:46–53. Doi: 10.1016/j.exger.2018.12.013.
28 Hwang C-L, Yoo J-K, Kim H-K, et al. Novel all-extremity high-intensity interval training improves aerobic fitness, cardiac function and insulin resistance in healthy older adults. *Exp Gerontol* 2016; 82:112–119. Doi: 10.1016/j.exger.2016.06.009.
29 Jung ME, Bourne JE, Beauchamp MR, et al. High-intensity interval training as an efficacious alternative to moderate-intensity continuous training for adults with prediabetes. *J Diabetes Res* 2015; 2015:191595. Doi: 10.1155/2015/191595.
30 Jung M, Locke S, Bourne J, et al. Cardiorespiratory fitness and accelerometer-determined physical activity following one year of free-living high-intensity interval training and moderate-intensity continuous training: A randomized trial. *International Journal of Behavioral Nutrition and Physical Activity* 2020; 17. Doi: 10.1186/s12966-020-00933-8.
31 Kargarfard M, Lam ETC, Shariat A, et al. Effects of endurance and high intensity training on ICAM-1 and VCAM-1 levels and arterial pressure in obese and normal weight adolescents. *Phys Sportsmed* 2016; 44(3):208–216. Doi: 10.1080/00913847.2016.1200442.
32 Kemmler W, Scharf M, Lell M, et al. High versus moderate intensity running

exercise to impact cardiometabolic risk factors: the randomized controlled RUSH-study. *Biomed Res Int* 2014; 2014:843095. Doi: 10.1155/2014/843095.
33 Khammassi M, Ouerghi N, Said M, et al. Continuous Moderate-Intensity but Not High-Intensity Interval Training Improves Immune Function Biomarkers in Healthy Young Men. *J Strength Cond Res* 2020; 34(1):249–256. Doi: 10.1519/JSC.0000000000002737.
34 Lanzi S, Codecasa F, Cornacchia M, et al. Short-term HIIT and Fat max training increase aerobic and metabolic fitness in men with class II and III obesity. *Obesity (Silver Spring)* 2015; 23(10):1987–1994. Doi: 10.1002/oby.21206.
35 Lazzer S, Tringali G, Caccavale M, et al. Effects of high-intensity interval training on physical capacities and substrate oxidation rate in obese adolescents. *J Endocrinol Invest* 2017; 40(2):217–226. Doi: 10.1007/s40618-016-0551-4.
36 Lunt H, Draper N, Marshall HC, et al. High intensity interval training in a real world setting: a randomized controlled feasibility study in overweight inactive adults, measuring change in maximal oxygen uptake. *PLoS One* 2014; 9(1):e83256. Doi: 10.1371/journal.pone.0083256.
37 Mitranun W, Deerochanawong C, Tanaka H, et al. Continuous vs interval training on glycemic control and macro- and microvascular reactivity in type 2 diabetic patients. *Scand J Med Sci Sports* 2014; 24(2):e69-76. Doi: 10.1111/sms.12112.
38 Nie J, Zhang H, Kong Z, et al. Impact of high-intensity interval training and moderate-intensity continuous training on resting and postexercise cardiac troponin T concentration. *Exp Physiol* 2018; 103(3):370–380. Doi: 10.1113/EP086767.
39 Nikseresht M, Hafezi Ahmadi MR, Hedayati M. Detraining-induced alterations in adipokines and cardiometabolic risk factors after nonlinear periodized resistance and aerobic interval training in obese men. *Appl Physiol Nutr Metab* 2016; 41(10):1018–1025. Doi: 10.1139/apnm-2015-0693.
40 Novaković M, Prokšelj K, Rajkovič U, et al. Exercise training in adults with repaired tetralogy of Fallot: A randomized controlled pilot study of continuous versus interval training. *Int J Cardiol* 2018; 255:37–44. Doi: 10.1016/j.ijcard.2017.12.105.
41 Nugent SF, Jung ME, Bourne JE, et al. The influence of high-intensity interval training and moderate-intensity continuous training on sedentary time in overweight and obese adults. *Appl Physiol Nutr Metab* 2018; 43(7):747–750. Doi: 10.1139/apnm-2017-0453.
42 Oh S, So R, Takashi S, et al. High-Intensity Aerobic Exercise Improves Both Hepatic Fat Content and Stiffness in Sedentary Obese Men with Nonalcoholic Fatty Liver Disease. *Scientific Reports* 2017; 7:43029. Doi: 10.1038/srep43029.
43 Paolucci EM, Loukov D, Bowdish DME, et al. Exercise reduces depression and inflammation but intensity matters. *Biol Psychol* 2018; 133:79–84. Doi: 10.1016/j.biopsycho.2018.01.015.
44 Raleigh JP, Giles MD, Scribbans TD, et al. The impact of work-matched interval

training on VO2peak and VO2 kinetics: diminishing returns with increasing intensity. *Appl Physiol Nutr Metab* 2016; 41(7):706–713. Doi: 10.1139/apnm-2015-0614.
45 Ramírez-Vélez R, Hernández-Quiñones PA, Tordecilla-Sanders A, et al. Effectiveness of HIIT compared to moderate continuous training in improving vascular parameters in inactive adults. *Lipids Health Dis* 2019; 18(1):42. Doi: 10.1186/s12944-019-0981-z.
46 Rognmo Ø, Hetland E, Helgerud J, et al. High intensity aerobic interval exercise is superior to moderate intensity exercise for increasing aerobic capacity in patients with coronary artery disease. *Eur J Cardiovasc Prev Rehabil* 2004; 11(3):216–222. Doi: 10.1097/01.hjr.0000131677.96762.0c.
47 Shing CM, Webb JJ, Driller MW, et al. Circulating adiponectin concentration and body composition are altered in response to high-intensity interval training. *J Strength Cond Res* 2013; 27(8):2213–2218. Doi: 10.1519/JSC.0b013e31827e1644.
48 Sperlich B, De Marées M, Koehler K, et al. Effects of 5 weeks of high-intensity interval training vs. volume training in 14-year-old soccer players. *J Strength Cond Res* 2011; 25(5):1271–1278. Doi: 10.1519/JSC.0b013e3181d67c38.
49 Stensvold D, Viken H, Steinshamn SL, et al. Effect of exercise training for five years on all cause mortality in older adults-the Generation 100 study: randomised controlled trial. *BMJ* 2020; 371:m3485. Doi: 10.1136/bmj.m3485.
50 Støa EM, Meling S, Nyhus L-K, et al. High-intensity aerobic interval training improves aerobic fitness and HbA1c among persons diagnosed with type 2 diabetes. *Eur J Appl Physiol* 2017; 117(3):455–467. Doi: 10.1007/s00421-017-3540-1.
51 Sun S, Zhang H, Kong Z, et al. Twelve weeks of low volume sprint interval training improves cardio-metabolic health outcomes in overweight females. *J Sports Sci* 2019; 37(11):1257–1264. Doi: 10.1080/02640414.2018.1554615.
52 Taylor J, Holland D, Iven Mielke G, et al. Effect of High-Intensity Interval Training on Visceral and Liver Fat in Cardiac Rehabilitation: A Randomized Controlled Trial. *Obesity* 2020; 28. Doi: 10.1002/oby.22833.
53 Tschentscher M, Eichinger J, Egger A, et al. High-intensity interval training is not superior to other forms of endurance training during cardiac rehabilitation. *Eur J Prev Cardiol* 2016; 23(1):14–20. Doi: 10.1177/2047487314560100.
54 van Biljon A, McKune AJ, DuBose KD, et al. Short-Term High-Intensity Interval Training Is Superior to Moderate-Intensity Continuous Training in Improving Cardiac Autonomic Function in Children. *Cardiology* 2018; 141(1):1–8. Doi: 10.1159/000492457.
55 Vella CA, Taylor K, Drummer D. High-intensity interval and moderate-intensity continuous training elicit similar enjoyment and adherence levels in overweight and obese adults. *Eur J Sport Sci* 2017; 17(9):1203–1211. Doi: 10.1080/17461391.2017.1359679.
56 Vogiatzis I, Terzis G, Nanas S, et al. Skeletal muscle adaptations to interval training in patients with advanced COPD. *Chest* 2005; 128(6):3838–3845. Doi: 10.1378/chest.128.6.3838.

57 Winn NC, Liu Y, Rector RS, et al. Energy-matched moderate and high intensity exercise training improves nonalcoholic fatty liver disease risk independent of changes in body mass or abdominal adiposity - A randomized trial. *Metabolism* 2018; 78:128–140. Doi: 10.1016/j.metabol.2017.08.012.
58 Zhang H, Nie J, Kong Z, et al. Impact of High-intensity Interval Exercise and Moderate-Intensity Continuous Exercise on the Cardiac Troponin T Level at an Early Stage of Training. *J Vis Exp* 2019; (152). Doi: 10.3791/60252.

2.2. *Equalization based on total exercise volume*

1 Alkahtani SA, Byrne NM, Hills AP, et al. Interval training intensity affects energy intake compensation in obese men. *Int J Sport Nutr Exerc Metab* 2014; 24(6):595–604. Doi: 10.1123/ijsnem.2013-0032.
2 Astorino TA, Schubert MM, Palumbo E, et al. Effect of two doses of interval training on maximal fat oxidation in sedentary women. *Med Sci Sports Exerc* 2013; 45(10):1878–1886. Doi: 10.1249/MSS.0b013e3182936261.
3 Buchheit M, Laursen PB, Kuhnle J, et al. Game-based training in young elite handball players. *Int J Sports Med* 2009; 30(4):251–258. Doi: 10.1055/s-0028-1105943.
4 Cardozo GG, Oliveira RB, Farinatti PTV. Effects of high intensity interval versus moderate continuous training on markers of ventilatory and cardiac efficiency in coronary heart disease patients. *ScientificWorldJournal* 2015; 2015:192479. Doi: 10.1155/2015/192479.
5 Chin EC, Yu AP, Lai CW, et al. Low-Frequency HIIT Improves Body Composition and Aerobic Capacity in Overweight Men. *Med Sci Sports Exerc* 2020; 52(1):56–66. Doi: 10.1249/MSS.0000000000002097.
6 Christensen RH, Wedell-Neergaard A-S, Lehrskov LL, et al. Effect of Aerobic and Resistance Exercise on Cardiac Adipose Tissues: Secondary Analyses From a Randomized Clinical Trial. *JAMA Cardiol* 2019; 4(8):778–787. Doi: 10.1001/jamacardio.2019.2074.
7 Ciolac EG, Bocchi EA, Bortolotto LA, et al. Effects of high-intensity aerobic interval training vs. moderate exercise on hemodynamic, metabolic and neuro-humoral abnormalities of young normotensive women at high familial risk for hypertension. *Hypertens Res* 2010; 33(8):836–843. Doi: 10.1038/hr.2010.72.
8 Corvino RB, Oliveira MFM, Denadai BS, et al. Speeding of oxygen uptake kinetics is not different following low-intensity blood-flow-restricted and high-intensity interval training. *Exp Physiol* 2019; 104(12):1858–1867. Doi: 10.1113/EP087727.
9 De Strijcker D, Lapauw B, Ouwens DM, et al. High intensity interval training is associated with greater impact on physical fitness, insulin sensitivity and muscle mitochondrial content in males with overweight/obesity, as opposed to continuous endurance training: a randomized controlled trial. *J Musculoskelet Neuronal Interact*

2018; 18(2):215–226.

10 Delextrat A, Martinez A. Small-sided game training improves aerobic capacity and technical skills in basketball players. *Int J Sports Med* 2014; 35(5):385–391. Doi: 10.1055/s-0033-1349107.

11 Gaesser GA, Wilson LA. Effects of continuous and interval training on the parameters of the power-endurance time relationship for high-intensity exercise. *Int J Sports Med* 1988; 9(6):417–421. Doi: 10.1055/s-2007-1025043.

12 Ghardashi Afousi A, Izadi MR, Rakhshan K, et al. Improved brachial artery shear patterns and increased flow-mediated dilatation after low-volume high-intensity interval training in type 2 diabetes. *Exp Physiol* 2018; 103(9):1264–1276. Doi: 10.1113/EP087005.

13 Gorostegi-Anduaga I, Corres P, MartinezAguirre-Betolaza A, et al. Effects of different aerobic exercise programmes with nutritional intervention in sedentary adults with overweight/obesity and hypertension: EXERDIET-HTA study. *Eur J Prev Cardiol* 2018; 25(4):343–353. Doi: 10.1177/2047487317749956.

14 Gosselin LE, Kozlowski KF, DeVinney-Boymel L, et al. Metabolic response of different high-intensity aerobic interval exercise protocols. *J Strength Cond Res* 2012; 26(10):2866–2871. Doi: 10.1519/JSC.0b013e318241e13d.

15 Hornbuckle LM, McKenzie MJ, Whitt-Glover MC. Effects of high-intensity interval training on cardiometabolic risk in overweight and obese African-American women: a pilot study. *Ethn Health* 2018; 23(7):752–766. Doi: 10.1080/13557858.2017.1294661.

16 Jaureguizar KV, Vicente-Campos D, Bautista LR, et al. Effect of High-Intensity Interval Versus Continuous Exercise Training on Functional Capacity and Quality of Life in Patients With Coronary Artery Disease: A RANDOMIZED CLINICAL TRIAL. *J Cardiopulm Rehabil Prev* 2016; 36(2):96–105. Doi: 10.1097/HCR.0000000000000156.

17 Jiménez-García JD, Martínez-Amat A, De la Torre-Cruz MJ, et al. Suspension Training HIIT Improves Gait Speed, Strength and Quality of Life in Older Adults. *Int J Sports Med* 2019; 40(2):116–124. Doi: 10.1055/a-0787-1548.

18 Kilit B, Arslan E. Effects of High-Intensity Interval Training vs. On-Court Tennis Training in Young Tennis Players. *J Strength Cond Res* 2019; 33(1):188–196. Doi: 10.1519/JSC.0000000000002766.

19 Lee C-L, Hsu W-C, Cheng C-F. Physiological Adaptations to Sprint Interval Training with Matched Exercise Volume. *Med Sci Sports Exerc* 2017; 49(1):86–95. Doi: 10.1249/MSS.0000000000001083.

20 Lira FS, Antunes BM, Figueiredo C, et al. Impact of 5-week high-intensity interval training on indices of cardio metabolic health in men. *Diabetes Metab Syndr* 2019; 13(2):1359–1364. Doi: 10.1016/j.dsx.2019.02.006.

21 Nytrøen K, Rolid K, Andreassen AK, et al. Effect of High-Intensity Interval Training in De Novo Heart Transplant Recipients in Scandinavia. *Circulation* 2019; 139(19):2198–2211. Doi: 10.1161/CIRCULATIONAHA.118.036747.

22 O'Leary TJ, Collett J, Howells K, et al. High but not moderate-intensity endurance training increases pain tolerance: a randomised trial. *Eur J Appl Physiol* 2017; 117(11):2201–2210. Doi: 10.1007/s00421-017-3708-8.
23 Pinto N, Salassi JW, Donlin A, et al. Effects of a 6-Week Upper Extremity Low-Volume, High-Intensity Interval Training Program on Oxygen Uptake, Peak Power Output, and Total Exercise Time. *J Strength Cond Res* 2019; 33(5):1295–1304. Doi: 10.1519/JSC.0000000000002008.
24 Racil G, Ben Ounis O, Hammouda O, et al. Effects of high vs. moderate exercise intensity during interval training on lipids and adiponectin levels in obese young females. *Eur J Appl Physiol* 2013; 113(10):2531–2540. Doi: 10.1007/s00421-013-2689-5.
25 Riiser A, Ripe S, Aadland E. Five training sessions improves 3000 meter running performance. *J Sports Med Phys Fitness* 2015; 55(12):1452–1458.
26 Rowan CP, Riddell MC, Gledhill N, et al. Aerobic Exercise Training Modalities and Prediabetes Risk Reduction. *Med Sci Sports Exerc* 2017; 49(3):403–412. Doi: 10.1249/MSS.0000000000001135.
27 Sosner P, Gayda M, Dupuy O, et al. Ambulatory blood pressure reduction following 2 weeks of high-intensity interval training on an immersed ergocycle. *Arch Cardiovasc Dis* 2019; 112(11):680–690. Doi: 10.1016/j.acvd.2019.07.005.
28 Terada T, Friesen A, Chahal BS, et al. Feasibility and preliminary efficacy of high intensity interval training in type 2 diabetes. *Diabetes Res Clin Pract* 2013; 99(2):120–129. Doi: 10.1016/j.diabres.2012.10.019.
29 Tsai H-H, Chang S-C, Chou C-H, et al. Exercise Training Alleviates Hypoxia-induced Mitochondrial Dysfunction in the Lymphocytes of Sedentary Males. *Sci Rep* 2016; 6:35170. Doi: 10.1038/srep35170.
30 Verbrugghe J, Agten A, Stevens S, et al. Exercise Intensity Matters in Chronic Nonspecific Low Back Pain Rehabilitation. *Med Sci Sports Exerc* 2019; 51(12):2434–2442. Doi: 10.1249/MSS.0000000000002078.
31 Yang M-T, Lee M-M, Hsu S-C, et al. Effects of high-intensity interval training on canoeing performance. *Eur J Sport Sci* 2017; 17(7):814–820. Doi: 10.1080/17461391.2017.1314553.

2.3. Equalization based on perceived exertion

1 Álvarez C, Ramírez-Vélez R, Ramírez-Campillo R, et al. Interindividual responses to different exercise stimuli among insulin-resistant women. *Scand J Med Sci Sports* 2018; 28(9):2052–2065. Doi: 10.1111/sms.13213.
2 Álvarez C, Ramírez-Campillo R, Ramírez-Vélez R, et al. Effects and prevalence of nonresponders after 12 weeks of high-intensity interval or resistance training in women with insulin resistance: a randomized trial. *J Appl Physiol (1985)* 2017; 122(4):985–996. Doi: 10.1152/japplphysiol.01037.2016.

3 Gremeaux M, Hannequin A, Laurent Y, et al. Usefulness of the 6-minute walk test and the 200-metre fast walk test to individualize high intensity interval and continuous exercise training in coronary artery disease patients after acute coronary syndrome: a pilot controlled clinical study. *Clin Rehabil* 2011; 25(9):844–855. Doi: 10.1177/0269215511403942.
4 Sandbakk Ø, Sandbakk SB, Ettema G, et al. Effects of intensity and duration in aerobic high-intensity interval training in highly trained junior cross-country skiers. *J Strength Cond Res* 2013; 27(7):1974–1980. Doi: 10.1519/JSC.0b013e3182752f08.
5 Schmit C, Duffield R, Hausswirth C, et al. Optimizing Heat Acclimation for Endurance Athletes: High- Versus Low-Intensity Training. *Int J Sports Physiol Perform* 2018; 13(6):816–823. Doi: 10.1123/ijspp.2017-0007.
6 Soh S-H, Joo MC, Yun NR, et al. Randomized Controlled Trial of the Lateral Push-Off Skater Exercise for High-Intensity Interval Training vs Conventional Treadmill Training. *Arch Phys Med Rehabil* 2020; 101(2):187–195. Doi: 10.1016/j.apmr.2019.08.480.
7 Wens I, Dalgas U, Vandenabeele F, et al. High Intensity Exercise in Multiple Sclerosis: Effects on Muscle Contractile Characteristics and Exercise Capacity, a Randomised Controlled Trial. *PLoS One* 2015; 10(9):e0133697. Doi: 10.1371/journal.pone.0133697.

2.4. *Equalization by other methods (same work duration for same mean intensity; same exercise relative volume)*

1 Blackwell J, Atherton PJ, Smith K, et al. The efficacy of unsupervised home-based exercise regimens in comparison to supervised laboratory-based exercise training upon cardio-respiratory health facets. *Physiol Rep* 2017; 5(17):e13390. Doi: 10.14814/phy2.13390.
2 Cavar M, Marsic T, Corluka M, et al. Effects of 6 Weeks of Different High-Intensity Interval and Moderate Continuous Training on Aerobic and Anaerobic Performance. *J Strength Cond Res* 2019; 33(1):44–56. Doi: 10.1519/JSC.0000000000002798.
